# Editorial: Bioresponsive nanomaterials for drug delivery or controlled release

**DOI:** 10.3389/fbioe.2023.1165782

**Published:** 2023-03-20

**Authors:** Yan Pan, Kezhen Qi, Jijun Fu, Wei Gao, Zhao Li, Zhiqiang Lin

**Affiliations:** ^1^ Department of Pharmacology, Institute of Systems Biomedicine, Beijing Key Laboratory of Tumor Systems Biology, School of Basic Medical Sciences, Peking University, Beijing, China; ^2^ College of Pharmacy, Dali University, Dali, Yunnan, China; ^3^ Key Laboratory of Molecular Target and Clinical Pharmacology, School of Pharmaceutical Sciences, The First Affiliated Hospital of Guangzhou Medical University and the Fifth Affiliated Hospital of Guangzhou Medical University, Guangzhou Medical University, Guangzhou, China; ^4^ Department of Pharmaceutical Sciences, College of Pharmacy, Rogel Cancer Center, University of MI, Ann Arbor, MI, United States; ^5^ Department of Hepatobiliary Surgery, Peking University People’s Hospital, Beijing, China

**Keywords:** bioresponsive nanomaterials, drug delivery, controlled release, targeting, novel nanomedicine

## Bioresponsive nanomedicine for precision drug delivery

Bioresponsive nanomedicines in response to the signals of biological signals, pathological stimuli, or external signals have attracted increasing attention from various researchers for precision drug delivery (Hong et al., 2023). Through targeting delivery or controlling the release of payloads in nanoparticles, these designs have greatly enhanced the efficacy as well as decreased the toxicity of existing drugs (Hong et al., 2023). In view of the significant progress in this field and the clinical translation prospect of many innovative nanomedicines, basic research needs to be enhanced. This Research Topic *Bioresponsive Nanomaterials for Drug Delivery or Controlled Release* focuses on new concepts, designs, methods of synthesis, preparation, and evaluation of nanomaterials. The objectives of this Research Topic were to provide insights into the design principles and evaluation methods of bioresponsive nanoparticles in the latest studies. Here, we present a Research Topic of five original articles in diverse nanomaterials for drug delivery and clinical evaluation of a microbubble contrast agent.

### Bioresponsive nanomaterials for synergistic or enhanced photothermal therapy

In recent years, nanomaterials have been exploited as a kind of carrier for enhanced cancer therapy owing to their distinctive advantages in drug delivery. Further, these nanoparticles are frequently designed for multifunctional therapeutical platforms that combine multiple therapeutical means for synergistic therapy (Lu et al., 2016). In this Research Topic, Leng et al. and Yao et al. describe different drug carriers for synergistic or enhanced photothermal therapy of breast cancer. Leng et al. have developed a tumor membrane coating nano-platform (PDA@MB) with photosensitizers and chemo drugs for photothermal therapy (PTT) combined with chemotherapy. It was able to avoid being captured by macrophages and enhanced tumor-targeting effect due to the camouflage of cell membranes. Under the irradiation of the NIR laser, PDA@MB produced high temperatures to rupture the coated membrane, causing drug release of CuB, which significantly improved the therapeutical outcomes (Leng et al.). Yao et al. have prepared injectable and temperature-sensitive hydrogel carriers with titanium carbide for photothermal therapy of breast cancer. Pluronic F127 was chosen as a thermosensitive material maintaining titanium carbide (Ti3C2) nanoparticles with excellent photothermal efficiency for localized photothermal therapy. It demonstrated the superior antitumor effect of this hydrogel system as well as satisfactory biocompatibility and biosafety (Yao et al.).

### Bioresponsive nanomaterials as vaccine adjuvants

As novel vaccine adjuvants, nanoparticle-based adjuvants have shown unique advantages and broad application prospects (Ma et al.). Besides the Al adjuvant which has been widely used in clinics for many years, other metal adjuvants like Mn, Zn, and so on have been developed over the years due to their unique advantages in boosting vaccines. In this Research Topic, Ma et al. report a manganese-based nanoadjuvant to improve the immunological effect of deoxyribonucleic (DNA) vaccines of influenza A (H5N1). This nanosystem was found to be capable of protecting DNA units, enhancing cellular uptake by macrophages, boosting activation of immune cells, and inducing both cellular and humoral immunity (Ma et al.).

### Bioresponsive nanomaterials for organelle targeting drug delivery

Cell function disorders relating to diseases are frequently reflected at the level of organelles. The development of organelle-targeting drug delivery systems is beneficial for improving disease diagnosis and therapeutic effects. Therefore, organelle-targeting drug delivery systems should be the next-generation precision medications. Li et al. designed a kind of curcumin/TPP-CZL nanomicelles targeting mitochondria in cancer cells. TPP-CZL nanomicelles enhanced the delivery of drugs that can finally target the mitochondria, significantly reducing the mitochondrial membrane potentials as well as activating apoptosis-related pathways in liver cancer cells. Therefore, the authors present a potential drug delivery system for efficiently targeting the mitochondria of liver cancer cells (Li et al.).

### Clinical evaluation of nanomaterials

While a great number of innovative nanomedicines or microparticles have been designed and published with ideal therapeutical efficacy in animal models, only a few are clinically available (Chen et al.). In this Research Topic, Chen et al. highlight the clinical applications of SonoVue, a clinically available microbubble contrast agent that can help surgeons to map lymphatic capillaries before the operation of thyroid carcinoma. Through clinical evaluation on SonoVue, the diagnostic accuracy was found to be 86.67% (for central lymph node metastasis) and 91.67% (for lateral compartment lymph node metastasis), respectively (Chen et al.). However, lymphatic contrast-enhanced ultrasound with SonoVue alone has limitations of false negatives in some specific cases, which need to be further improved in the future. We hope this Research Topic will encourage new ideas for the design of novel nanomaterials as well as clinical evaluations of nanomedicines.
